# Supplementary material for: Are carbon emissions trading and green financial instruments synergistic? -Comprehensive quantitative research based on content analysis
Source: PLoS One. 2024 Mar 7;19(3):e0298601. doi: 10.1371/journal.pone.0298601 (PMC10919601; doi:10.1371/journal.pone.0298601)
Supplement: S1 Data — (ZIP) [file pone.0298601.s001.zip › Data/地级市Z_F工作报告.zip!/┴╔─■/┤≤┴1⁄4╩╨/└┤╫╘╣1⁄2╓┌║┼í╛┬φ┐╦╩2╛▌═°í┐_2015.docx]

各位代表：

　　现在，我代表市政府向大会报告工作，请予审议，并请市政协委员和其他列席人员提出意见。

　　一、2014年政府工作回顾

　　过去的一年，在省委、省政府和市委的正确领导下，市政府全面贯彻党的十八大和十八届三中、四中全会及习近平总书记系列重要讲话精神，团结带领全市人民积极应对错综复杂的经济形势，坚持稳中求进的总基调，统筹推进稳增长、促改革、调结构、惠民生、防风险各项工作，全市经济社会呈现良好的发展态势。

　　（一）经济实现平稳增长。

　　预计实现地区生产总值8000亿元以上，增长6%左右，人均18760美元。固定资产投资6800亿元，增长5%左右；社会消费品零售总额增长12%左右；外贸自营出口与上年持平；一般公共预算收入780.8亿元，下降8.2%；规模以上工业企业利税、利润分别增长10%和15%；城镇登记失业率控制在2.65%；居民消费价格涨幅为2%，城乡居民可支配收入分别增长9.2%和10.5%；万元GDP能耗下降3.8%。虽然经济发展出现波动，但实体经济发展平稳，就业、物价始终保持稳定，经济运行处在合理区间。

　　（二）结构调整取得新进展。

　　传统产业加快转型升级。石化、装备制造、造船、电子信息等优势产业集群化发展，产业链条不断延伸，长兴岛石化产业基地纳入国家石化产业规划布局，炼化一体化项目有序推进；东风日产汽车、比亚迪电动客车整车下线，汽车制造业产值增长40%，汽车及零部件产业集群实现产值600亿元；嘉盛新材料、铭源石化等一批投资超10亿元重大项目投产。全市完成工业投资2200亿元，增长9.3%；19个省重点产业集群销售收入全部超过百亿元，其中3个超过千亿元。自主创新能力提升，助推传统产业向中高端迈进，开展38项重大关键技术攻关，取得了一批重大成果；组建了10个产业技术研究院，新增1个国家级高新技术产业化基地和两个火炬特色产业基地；我市有9项科技成果获得国家科学技术奖励，其中大连化物所甲醇制取低碳烯烃技术获得国家技术发明一等奖。全市发明专利授权量1363件，完成技术交易额110亿元。

　　战略性新兴产业形成了多极支撑格局。智能装备、新能源、新材料等9个重点产业蓬勃发展，大连机床、光洋科技等企业研制的产品进入航空航天、核工业和军工领域，国产首台三代核电反应堆压力容器制造完成，比克电池大连产业项目投产。海洋工程装备产值增长95%。集成电路发展势头良好，产业规模达70亿元。

　　服务业拉动作用增强。实施了加快服务业发展四年行动计划，从工业企业中分离各类生产性服务企业390户；电子商务长足发展，大连再生资源交易所、东北亚现货商品交易所等16家大宗商品电子商务平台快速崛起；软件和信息服务业实现销售收入1485亿元，增长10%。全年共接待游客5846万人次，实现旅游业总收入1065亿元，增长18.2%；主城区总部经济、楼宇经济规模不断壮大，新兴服务业态不断出现。全市服务业增加值占地区生产总值43.9%，同比提高了1个百分点。

　　都市型现代农业加快发展。战胜50年一遇的旱灾，农业减产不减收。新增设施农业10万亩，新建海洋牧场10万亩，新发展农民专业合作社和家庭农场100家，加快推进10个都市型现代农业园区和9个农产品加工重点项目，国家级现代农业示范区通过农业部评审。

　　非公经济持续壮大。实施中小微企业成长工程，发展各类企业27310户、注册资本1333.4亿元，分别增长69.9%和113%。出台小微商贸企业专项贷款扶持政策，为700户小微商贸企业授信贷款7.56亿元。开展中小企业融资服务年活动，全年发放风险贷款9.4亿元，新增小微企业信贷余额65亿元。重点培育省级创新型中小企业100家，中小企业公共服务平台达到26个，民营经济增加值增长9%。

　　（三）改革开放进一步深化。

　　行政管理体制改革深入推进。进一步简政放权，全年取消、下放和调整619项行政审批事项，全面落实国务院、省政府462项下放职权“接放管”工作。推进投资审批、建设工程审批等专项改革，优化办事流程，提高行政审批效率，审批时限缩短15%。深化工商登记制度改革，31项前置审批事项改为后置审批。完成食品药品监管体制、卫生与计生机构改革，整合资源提升公共服务及市场监管水平。出台政府购买服务实施办法，确保具备条件的社会力量平等参与社会治理和服务。

　　经济体制改革加快实施。出台深化国资国企改革意见，推进股权多元化改革，市属国有企业利润总额增长5%。发展多元化市场主体，制定非公有制经济市场准入负面清单和发展混合所有制经济政策，全年民间投资增长2.4%。

　　统筹城乡改革发展。甘井子区综合配套改革不断深化。建立了中心镇建设引导基金等融资机制。完成10个乡镇、15个村土地承包经营权确权登记颁证试点工作，集体林权确权100%。推进户籍制度改革，建立了城乡统一的户口登记制度。加强社区基础工作，政府管理与群众自治相互衔接。

　　开放型经济新体制逐步建立。以开放促改革，充分发挥外资项目的技术溢出和综合带动效应。全年外商投资注册资本25亿美元、到位内资2580亿元，投资高端制造业、现代服务业项目占比超过90%。核准境外投资项目99个、协议投资增长15%，完成9个海外科技企业并购，机床集团继并购美、德企业后又在俄罗斯建厂，瓦轴美国工程技术中心挂牌运行，大连机车获得了南非232台内燃机车订货合同。出台24条贸易便利化举措，复制上海自贸区5项快速通关措施，完成外贸自营出口295亿美元。

　　（四）全域城市化加快推进。

　　“三个向北”战略深入实施。统筹城乡规划，推进“多规合一”试点，形成城乡一体化发展格局。加快推进基础设施建设，城乡公共服务水平不断提升。实施175项重大基础设施项目建设，南部滨海大道、旅顺中部通道建设加快推进，地铁1、2号线开通试运行，202路轻轨延伸线载客试运营，长山大桥、皮口陆港客运中心、大伙房水库输水入连等一批工程竣工，新机场、丹大快铁、渤海大道等工程加快建设，较为完善的全域路网、水网、电网布局基本形成，城市发展框架全面拉开，为“十三五”发展奠定了坚实基础。

　　城市核心功能不断完善。国际航运中心和物流中心协调发展。太平湾港产城一体化建设顺利推进，庄河港扩建工程有序展开。全市港口完成货物吞吐量4.2亿吨、集装箱吞吐量1013.2万标箱；完成海铁联运32万标箱，增长10.3%。社会物流价值总额2.4万亿元，实现物流业增加值900亿元。区域性金融中心建设取得新进展。大连商品交易所功能进一步增强，交易品种增至16个，上线夜盘品种8个。建立科技金融创新服务体系，组建大连交易（所）集团和大连港投融资控股集团，助推4家企业香港上市、22家企业“新三板”挂牌，形成多层次资本市场发展格局。辽宁忠旺财务公司、浦发银行离岸金融总部、东北亚石化交易中心在连设立。全年新增信贷与融资2638亿元，资本市场直接融资增长75%。跨境贸易人民币结算增长101.3%，占全省的55.2%。

　　“四大组团”发展活力增强。金普新区获批国家级新区，总体规划编制基本完成，先行先试政策陆续落地。普湾新区开发建设初具形象。金州新区开放功能不断增强，产业丰厚度不断提升，智能装备产业集群成为国家新型工业化产业示范基地。保税区保税业务发展势头良好，“三城联创”初见成效。坚持产城融合，加快重点区域开发建设，旅顺经济技术开发区实现新发展，长兴岛石化产业基地中石油、恒力等重大炼化一体龙头项目全面启动，瓦房店国内最大的轴承电子交易平台投入运行，庄河循环经济示范基地建设进展顺利，花园口集聚新兴产业项目近百个，长海现代海洋牧场和国际旅游避暑胜地建设取得良好成效。沿海经济带19个重点产业园区财政收入、固定资产投资和利用外资分别占全市的39.1%、75%和80.1%，对全市经济支撑作用进一步增强。

　　（五）城市治理水平不断提升。

　　城市建设管理得到加强。树立系统治理的理念，针对城市建设和管理中的突出问题，实施市容环境集中整治，无序设摊、占道停车、工地扬尘等9类群众反映强烈、影响城市形象的问题得到了较好解决。实施城建“五个一”工程69项、“三个一”工程10项，新建停车泊位20300个，维修主次干道17条、街巷路146条，新增、调整公交线路51条，中心城区179公里健身步道连接成网。智慧城市建设稳步推进，我市入选国家首批“宽带中国”示范城市和信息惠民试点城市。

　　城乡生态环境不断改善。实施污染减排和大气、水环境、海岸带综合整治工程，主要污染物平均减排2%以上，农村垃圾、生活污水处理率分别达到39%和43%。全年植树造林60.3万亩。经过4年的青山生态系统工程建设，我市林木绿化率达到44.5%。开展农村环境连片整治，旅顺龙河治理、庄河三河入海口生态绿化、花园口水系综合治理加快推进。持续推进“六化”工程建设，新建通屯油路876公里，宜居乡村建设取得了新成效。

　　社会治理创新进一步深化。整合行政审批、行政投诉、公共资源交易等服务管理职能，搭建开放型、一站式社会公共服务平台，建立市民综合服务、矛盾纠纷化解、社会组织孵化、平安大连建设四大工作体系，社会治理能力大幅提升。落实依法逐级走访制度，信访秩序进一步好转。开展了社会治安专项治理，严厉打击了各种违法犯罪活动。组建公安“蓝鲨机动队”，应急处理和反恐快速反应能力显著增强。建立“党政同责、一岗双责”安全责任体系，135个单位获得“全国安全社区”称号，我市成为全国安全发展试点城市。

　　（六）民生社会事业持续改善。

　　加大民生社会事业财政投入。全年用于教育、文化、卫生、体育、社会保障等财政支出686.2亿元，占一般公共预算支出的69.3%，高于一般公共预算支出增幅4.3个百分点。15项重点民生工程高质量按时完成。

　　社会保障水平显著提升。全年实现城镇实名制就业22.5万人，近5年城镇登记失业率一直控制在3%以内。新农合最低筹资标准由5年前的140元提高到520元；企业退休人员基本养老金月均2150元，居全省第一；城乡低保标准分别比5年前提高58.3%和103.6%；全市327万人享受职工医保门诊统筹待遇，支付医保统筹基金超10亿元。实施住房维修“一二三”工程，完成“暖房子”工程300万平方米，改造棚户区2.4万平方米，维修弃管房20万平方米，新增保障家庭公租房11788套。华铜矿塌陷区搬迁安置工程有序推进，城子坦淹没区搬迁安置工程基本完成。

　　社会事业不断进步。义务教育均衡发展水平居全国前列，中小学公用经费标准保持全省领先，就读省示范性高中学生比例达46%，新增公办幼儿学位3080个，三大职教集团和职教基地一期工程建设进展顺利，高校与地方合作取得新成果。医疗软实力提升工程成效明显，投入23亿元建设了新的市卫生中心，市妇儿医疗中心开诊营业，儿童医院改扩建工程快速推进。积极稳妥推进“单独两孩”政策，全市人口自然增长率稳定在3.36‰。文化体育事业协调发展，国家公共文化服务示范区建设稳步推进，普湾新区“三馆”、大连文化产业创新园区和中心书城加快建设，在全省率先实现广播电视信号全覆盖。全民健身运动丰富多彩，我市获得第十二届省运会团体冠军。

　　（七）政府自身建设全面加强。

　　转变作风成效明显。以“钉钉子”精神推进第一批党的群众路线教育实践活动整改方案落实，对89个单位“四风”整治进行专项督查。扎实开展第二批教育实践活动，“工作落实年”活动取得显著成效。建立政府督查可视系统，及时跟踪督办重点工程建设。我市在全省政府系统综合绩效考评中再次荣获第一名，实现“十二连冠”。

　　依法行政深入推进。加强法治政府建设，进一步优化行政决策程序；坚持重大事项决策前向人大报告、与政协民主协商，认真落实人大专题询问、执法检查和政协专题协商意见；积极回应人大代表、政协委员对社会热点问题的关注，办结省市两级议案、建议、提案1385件；自觉接受人大法律监督、工作监督和政协民主监督以及社会各界的监督。

　　正风肃纪取得良好实效。积极落实中央巡视组和审计部门反馈意见，认真组织整改。全面落实中央八项规定，加强“四风”专项整治，强化行政监察和经济责任审计，坚决查处各种违法违纪案件。规范公用经费管理，财政预决算和“三公”经费信息全部公开，全年“三公”经费下降21%，公务支出和经费管理步入规范化、制度化、法制化的轨道。

　　民族宗教、残疾人、外事、侨务、对台、人防、档案、防震减灾、仲裁、双拥共建、民兵预备役、边海防等工作都取得了新进展。

　　各位代表，2014年大连经济社会发展成绩来之不易，凝聚了社会各界的心血、智慧和汗水，全市人民付出了艰辛的努力。在此，向给予政府工作大力支持的人大代表和政协委员，向各民主党派、工商联、无党派人士和人民团体，向离退休老同志，向中省直各部门和兄弟省市驻连机构，向驻连部队和武警官兵，向所有关心支持大连经济社会发展的海内外各界朋友表示衷心的感谢并致以崇高的敬意！

　　回顾过去的一年，我们必须清醒地看到，我市老工业基地的体制机制障碍依然存在，市场配置资源的决定性作用还没有充分发挥出来；经济结构不够合理，产业层次不够高，传统产业转型升级任务仍很艰巨；企业生产成本升高，融资渠道不够畅通，发展的软环境有待优化；税收增长乏力，还债节点集中，财政收支压力较大；城市快速建设与生态环境承载力的矛盾日益突出，城市综合管理的科学化水平有待提高，与群众期盼存在一定差距；政府部门少数人员作风不实、责任意识不强，以改革创新的思维破解难题、寻求发展的办法不多等等。对此，我们将高度重视并在今后的工作中认真加以解决。

　　二、2015年预期目标和主要任务

　　2015年是全面深化改革的关键之年，是全面推进依法治市的开局之年，也是全面实施“十二五”规划的收官之年。

　　我国经济发展正处于增长速度换挡期、结构调整阵痛期、前期刺激政策消化期“三期叠加”阶段。我市经济发展的外部环境和内在条件发生一系列趋势性变化，呈现了速度放缓、结构优化、动力转换三大特点，经济发展正在向形态更高级、分工更复杂、结构更合理的阶段演进。我们要适应新常态，应对新挑战，把握新机遇，求得新发展。当前，国家正在实施新一轮东北振兴战略，出台了一系列支持政策，在重大产业布局上向老工业基地倾斜，批准长兴岛建设世界级石化产业基地，这必将为我市加快产业结构调整步伐提供有利条件。国家实施“一带一路”战略，我市地处东北亚区域中心，是“一带”的桥头堡和“一路”的延伸点，国家级金普新区获批，彰显了大连对外开放的新优势，我们迎来大开发、大开放的历史性机遇。与此同时，我市又成为国家新型城镇化综合试点城市，要求在经济、文化、社会、生态等多个领域先行先试，这为全面深化改革、创新体制机制提供了强大动力。面对新的机遇和挑战，我们要更加注重深化改革和扩大开放，释放市场活力；更加注重提高自主创新能力，促进提质增效；更加注重提升公共服务，增进民生福祉；更加注重防范和化解风险，保持经济平稳运行。只要我们把握大势、坚定信心、保持定力、积极作为，就一定能够圆满完成市委确定的各项目标任务。

　　今年政府工作的总体思路是：全面贯彻党的十八大和十八届三中、四中全会及中央、省经济工作会议精神，按照市委十一届八次全会的要求，坚持稳中求进工作总基调，以提高经济质量和效益为中心，以改革开放创新为动力，以增进民生福祉为目标，以法治政府建设为保障，坚持四化统筹、协调推进，全面落实稳增长、促改革、调结构、惠民生、防风险各项任务，促进经济平稳健康发展和社会和谐稳定，为加快建设产业结构优化的先导区和经济社会发展的先行区而不懈奋斗。

　　经济社会发展的主要预期目标是：地区生产总值增长6.5%左右，固定资产投资增长7%左右，社会消费品零售总额增长13%左右，外贸自营出口增长3%左右，一般公共预算收入实现正增长，其中市本级一般公共预算收入与GDP同步增长，城乡居民可支配收入分别增长9%和10%，万元GDP综合能耗下降3.77%，城镇登记失业率控制在3%以内，居民消费价格涨幅控制在3%左右。

　　（一）着力推动经济健康发展。

　　更好地发挥消费的基础作用。多措并举促进大众消费，瞄准潜力大、前景好的消费热点，顺应多层次、个性化、多样化的消费趋势，激发养老、健康、家政、旅游、信息、住房、教育、文化、体育和绿色十大领域的消费需求。大力推进网上消费，改善网销环境，丰富促销活动，培育“假日经济”。努力提升居民消费能力，进一步增加中低收入居民收入，继续提高企业退休人员基本养老金和城乡居民基本养老保险基础养老金待遇水平；继续提高优抚对象抚恤和生活补助、城乡低保和农村五保对象补助水平，调整最低工资标准，同比例提高城、乡居民基本医保财政补助标准。

　　更好地发挥投资的关键作用。多管齐下扩大有效投资，加大基础设施和重大产业项目的建设力度。保持财政投资规模不减，确保“十二五”规划确定的城市建设目标的实现。启动一批新的重点项目，注重调结构、补短板、惠民生，增加公共产品的有效供给。强化央企在连投资的拉动作用，用股权、基金、债券等手段拓展投资渠道，采取BOT、BOO、PPP等方式推进项目建设，有效释放社会投资的潜力。全年安排亿元以上建设项目920个，总投资1.76万亿元。

　　更好地发挥出口的支撑作用。多元培育出口市场，完善鼓励出口政策，加强出口基地建设，优化出口结构，拓展国际市场，培育一批有实力的出口企业，形成外贸竞争新优势。大力发展服务贸易，积极培育出口品牌和境外营销网络，发展跨境电子商务，争取在我市设立国家跨境电子商务综合试验区。实施“走出去”战略，鼓励有条件的企业跨国（境）发展。

　　更好地发挥政策的引导作用。多方施策确保经济平稳运行，对企业技术改造、电子商务、参加展会、开拓市场及“走出去”等给予政策支持，破解企业发展“瓶颈”。强化生产要素保障，确保企业生产对能源、土地、资金、供气、供水的需求，引导要素向重点项目、优势企业、新兴产业倾斜配置。全年新增贷款和融资2500亿元，帮助中小企业融资400亿元以上，缓解企业融资难、融资贵问题。

　　（二）着力调整优化产业结构。

　　推动传统产业转型升级。加快工业化与信息化融合发展，落实加速折旧等措施，促进企业技术改造和创新产品、创新管理、创新商业模式，推动制造业向系统集成和制造服务化转型，让传统产业“老树开新花”。积极推动国家智能制造产业基地、国家高端轴承高新技术产业基地建设，实施一批重点领域的智能制造项目，加快工业机器人研制及应用。提升数控技术及装备、大型成套装备及集成等产业竞争优势。加大海洋工程、特种船舶等研发设计及市场开发力度。加快长兴岛石化产业基地建设，加速推进中石油、恒力炼化一体化、碧科烯烃等重大项目建设，拉长石化产业链条。推动东风日产、奇瑞汽车和比亚迪电动车、华晨专用车尽快达产，力争汽车产业集群达到千亿级规模。

　　做大做强战略性新兴产业。积极推动高端装备、集成电路、软件和信息服务、新能源汽车、新材料、生物医药等产业快速发展，强化政策引导，搞好要素保障，加大引资引智力度，促进集群发展，使之早日成为主导产业。支持华信云计算公共服务平台、腾讯互联网创新平台、北斗卫星应用产业园建设，打造“中国软件名城”。高新技术产业增加值占规模以上工业增加值的比重提升到60%以上。加快建筑产业化进程。

　　优先发展现代服务业。促进服务业与工农业协同发展、跨界发展、创新发展，坚持生产性服务业与生活性服务业并重，现代服务业与传统服务业并举，实现服务业量的扩大、质的提升。顺应港口城市产业一体化、航运交易金融一体化、物流物联集疏一体化发展趋势，牢牢抓住提质增效这个核心，以建设质量效益港、商务便利港、物流枢纽港、绿色低碳港为抓手，提高服务层级，完善枢纽功能，延伸港口价值链，主动向以绿色化、智慧化为特征的第五代港口转型升级，成为现代高端服务功能融合发展的重要载体。以国家级物流产业基地建设为牵动，全力推进太平湾港产城一体化发展。支持大连机场集团拓展业务，做大货邮转运规模。大力发展商贸物流、冷链物流、电子商务和邮政快递。加大区域性金融中心建设力度，发挥大连商品交易所的旗舰作用，积极支持在连金融机构拓展业务，进一步集聚功能性金融机构，大力发展互联网金融、航运金融、科技金融，推动多层次资本市场和现代保险服务业加快发展，全年新增金融机构10家以上，新增上市和挂牌企业20家以上。促进大连再生资源交易所、东北亚现货商品交易所、泰德煤网和北良国际农副产品交易中心做大做强。支持大商集团电商平台建设，叫响品牌，扩大覆盖，实现线上线下融合发展。规划建设辐射东北的特色超大型批发市场，提升商贸业品质，使大连成为东北地区的购物中心。完善金石滩国家级旅游度假区功能，推进长山群岛旅游度假区建设，不断推出新的旅游产品，丰富旅游内涵，使大连成为中国北方旅游胜地。

　　加快转变农业发展方式。加强国家现代农业示范区建设，大力发展渔业、蔬菜、畜牧、水果、花卉五大优势特色产业，新发展设施农业4万亩、海洋牧场10万亩，加快农业现代化进程。推进农田水利设施建设，完成水土流失治理34万亩，提高抗旱防灾能力。深化农村改革，完成300万亩土地确权登记颁证任务，积极培育新型农业经营主体，推进农村土地承包经营权流转，开展永久基本农田划定工作。

　　（三）着力保障增进民生福祉。

　　加大民生事业投入力度。坚持守住底线、突出重点、完善制度的基本思路，多些雪中送炭，更加注重保障基本民生，更加注重改善低收入群众生活，更加注重社会大局稳定。把更多的财力投入民生事业，高质量实施15项重点民生工程，确保全年民生支出高于上年水平，让广大人民群众共享改革发展成果。

　　积极促进充分就业。把充分就业作为经济社会发展的优先目标，积极破解结构性就业矛盾，实现城镇实名制就业21万人。重点做好高校毕业生、农村转移劳动力、不稳定就业群体、就业困难人员、退役军人的就业工作，大力开展就业和岗位技能培训，培训规模要达到3.6万人，确保“零就业”家庭至少有1人就业。制定应对可能出现的结构性失业预案，发挥市场作用，鼓励创业带动就业，实施高校毕业生就业促进计划和创业引领计划，年内完成创业培训3000人。提高公共就业服务能力，为劳动者求职提供及时便捷的服务，加强舆论引导，形成劳动光荣、崇尚技能的社会环境。

　　完善社会保障和救助体系。启动全民参保登记计划，进一步扩大城乡基本养老、医疗、失业、工伤、生育保险的覆盖面。推动全民基本医保由“扩大范围”向“提升质量”转变，新农合最低筹资标准提高到580元。按照“一个统一、五个同步”的要求，推进机关事业单位养老保险制度改革，建立与城镇职工统一的养老保险制度。完善临时救助工作体系，逐步建立“一门受理、协同办理”的救助机制。推动老龄事业与健康产业互动发展，完善养老服务体系，鼓励社会资本投资养老服务产业。加快推进残疾人小康进程，为残疾人提供更好的个性化服务。

　　推进保障性安居工程建设。城镇享住保障房家庭要覆盖20%左右，新开工建设或预购保障房2万套，基本具备入住条件9500套。保障性住房逐步从建房为主转向建设和租赁补贴并重。落实住房公积金惠民政策，新增公共租赁住房货币补贴5000户。改造旧城区17万平方米，完成城市棚户区（危旧房）改造8000套。启动住房“绿色建筑”行动，完善新型物业管理体制。继续实施住房维修“一二三”工程，完成“暖房子”工程300万平方米。实施城市居民室内供水旧管网改造工程。维修100条街巷路。加快农村危旧房改造，帮助农村贫困家庭解决生产和生活困难。

　　（四）着力推进全域城市化。

　　继续推进全域一体化基础设施建设。加快建设覆盖城乡的重大交通基础设施，推进渤海大道一期工程、金普城际铁路、丹大快铁、旅顺中部通道和陆岛交通体系建设，建成通屯油路800公里。完善全域一体化的供电、供水、供暖、网络等公共服务体系。

　　坚持产城融合发展。加速沿海经济带19个重点产业园区建设，按照特色化、集群化的要求，吸引新兴优势产业集聚，促进资源要素在“四大组团”合理配置，优化生产力布局。提升主城区辐射功能，发挥新市区开发带动作用，推动北黄海经济区加快发展。

　　大力推进卫星城和中心镇的规划建设。紧紧抓住我市作为国家新型城镇化综合试点的机遇，积极争取国家资金、政策的支持，大力推进瓦房店、长兴岛、普兰店、庄河、花园口、长海等卫星城的规划建设；大力推进太平湾、复州城、老虎屯、安波、皮杨、莲山、青堆、大郑、黑岛、獐子岛、广鹿岛等中心镇的规划建设。把促进卫星城和中心镇建设与加快产业发展结合起来，加大产业招商力度，提升产业支撑力，鼓励周边企业迁入中心镇工业园区，提高土地节约集约利用水平；把促进卫星城和中心镇建设与人口集聚结合起来，深化户籍制度改革，注重写好“人”字，有序推进农业转移人口市民化，不断提高城市化率；把促进卫星城和中心镇建设与完善社会保障结合起来，健全配套政策，提升城镇居民保障能力。加强外来人口的服务管理，对不具备落户条件的就业人口，要以居住证为载体，提供基本公共服务。

　　破解建设资金和土地资源难题。坚持“两条腿”走路，拓宽多元融资渠道，政府要加大投入力度，通过发债等方式筹集资金。用好政策性、开发性金融等工具，建立透明规范、可持续的城市建设融资机制。鼓励社会资本参与城市供水供气、公共交通、污水处理等设施的建设和经营。探索土地资源优化配置方式，保证城镇化对土地的需求。

　　（五）着力加快发展社会事业。

　　继续推进义务教育均衡发展。坚持立德树人，促进教育公平。实施校群、区际组团办学，促进优质教育资源向涉农地区和城市薄弱学校延伸。实施100所义务教育标准化学校建设，开展中小学教育质量综合评价改革试点，创评20所辽宁省课程改革示范校。适应高考改革新变化，促进高中教育优质特色发展。加快发展现代职业教育，优化布局，整合资源，推进职业教育结构调整，强化中高职的相互衔接，加快普湾新区职业教育基地建设。支持和引导民办幼儿园普惠性发展。继续实施学校食堂建设和农村中小学供暖设施改造工程，启动农村新招聘教师集体宿舍建设。支持在连高校内涵发展，充分发挥高校人才培养、科技创新、文化传播、产业推动的重要作用。

　　加快医疗卫生事业发展。以提升医疗软实力为牵动，开展名医、名科、名院“三名工程”，不断增强诊疗救治能力。推进医疗、医保、医药“三医联动”的医药卫生体制改革，着力解决医疗资源不均衡、人才供给不足的“瓶颈”问题。降低药物流通成本，逐步实现药品零差价。优化城乡医疗卫生格局，构建基层首诊、双向转诊、急慢分治、上下联动的分级诊疗制度，推进医保异地联网结算，方便就医群众。加快建立疾病应急救助制度，完善重特大疾病保障机制。推进中医药事业发展，鼓励社会资本兴办医疗、康复机构，引入国内优质医疗资源，探索与国外医疗机构合作办医方式，满足多层次医疗服务需求。坚持计划生育基本国策，出生人口自然增长率保持在3.2‰左右。

　　激发文化创造活力。深入实施公民道德建设工程，培育市民社会主义核心价值观，大力发掘和弘扬城市精神，提高城市文明程度和市民综合素质。推进大连图书馆新馆、大连历史博物馆等大型文化设施项目建设，完善现代公共文化服务体系，培育一批特色文化艺术品牌。继续实施文化惠民工程，全年组织公益演出400场以上。完善鼓励政策，引导民营资本与文化资源开发相结合，推进文化事业与文化产业融合发展。支持高新区动漫产业基地、旅顺影视基地建设，积极打造文化创意、动漫游戏、影视传媒和文化旅游产业集聚区。保护利用好小珠山、太阳沟等历史文化资源。完善文化创新环境，打造常演常新的旅游剧目。

　　推动体育事业健康发展。落实全民健身计划，实施体育惠民工程，提高竞技体育发展水平，出台促进体育产业发展配套政策，大力发展足球事业。

　　（六）着力提升城市建管水平。

　　大力推进城市基础设施建设。按照“品质立市”和建设现代化国际城市的要求，坚持高起点规划、高标准建设城市基础设施，提升城市承载力。加快推进地铁1、2号线的试运和验收工作，确保按期投入运营。启动地铁4、5号线和大连湾海底隧道前期工作，拓宽改造疏港路，实现南部滨海大道竣工通车。加快构建以轨道交通为骨干、快速公交和常规公交为主体、出租车为补充的现代都市公交体系。加快新机场和长海机场扩建工程建设，推进钻石海湾和新机场商务区基础设施建设。

　　加快智慧城市建设步伐。按照智能运行的发展理念，依托新一代信息技术，以信息化网络普及为载体，以智能化管理为手段，加快构筑信息资源共享、城市运转高效、公共服务便捷的智慧之城。重点推进智慧平台资源整合，实施智慧口岸、智慧交通、智慧城管、智慧教育、智慧卫生建设，加快4G业务发展及城乡光纤到户改造。推进电子政务云平台建设。

　　更加注重城乡综合治理。优化城市管理体制机制，推进城市网格化、精细化管理，促进规划、城建、综合执法、公安、交通、工商联合互动，着力解决马路停车、渣土撒漏、背街路巷脏乱等问题。借鉴国内外先进经验，强化城市车辆管理，运用市场机制加大城市停车场建设力度，逐步解决市民停车难问题。全力推进大连湾、梭鱼湾、泉水河二期3座污水处理厂和中心城区垃圾焚烧发电二期工程建设。加大海洋环境监测力度，加强海洋生态修复。实施生态景观绿化、矿坑环境治理等十大工程，全年植树6000万株，造林补植28万亩。加强饮用水源环境保护，提高水生态文明建设水平。实施大气污染防治行动计划，下大气力提升城市环境质量，单位工业增加值综合能耗同比降低3%，主要污染物平均减排2%以上，建设天蓝山绿水净的美丽大连。

　　（七）着力加强社会治理创新。

　　创新社会服务管理体系。坚持系统、依法、综合、源头治理的理念，以网格化管理、社会化服务为方向，完善以市民综合服务中心为主渠道的市、区、街道和社区四级综合服务管理工作体系，实行一个平台运行、一个流程办理、一套机制考核，及时反映和协调人民群众各方面利益诉求。加强社区规范化建设，强化社区为民服务功能。深化社会组织管理创新，引导社会组织参与社会管理和公共事务。加强和改进民族、宗教工作。

　　创新有效预防和化解社会矛盾机制。健全畅通有序的诉求表达、心理干预、矛盾调处、权益保障机制。坚持法定途径优先原则，解决信访突出问题。推进诉调对接、医患纠纷调解、物业管理纠纷化解3个中心建设，加快构建矛盾纠纷化解工作的新格局。

　　深入开展平安大连建设。健全立体化社会治安防控体系，依法打击各类违法犯罪活动。完善危机预防和管理体系，提高对自然灾害、事故灾难、公共卫生和社会安全事件的应急处置能力。全面落实安全生产责任制，加大隐患排查治理和安全防控力度，推进安全环保职业健康管理体系建设，提升企业本质安全水平。实施食品药品安全惠民工程，健全从生产加工到流通消费的全过程监管机制和社会共治制度。

　　高标准编制国民经济和社会发展第十三个五年规划。继续做好外事、侨务、对台、人防、档案、防震减灾、仲裁、双拥共建、民兵预备役和边海防等各项工作。

　　（一）全面深化改革，激发市场活力。认真贯彻党的十八届三中全会精神，落实市全面深化改革领导小组提出的各项改革任务。进一步理顺政府与市场的关系，以深化行政审批制度改革为突破口，协同推进各领域改革，最大限度地激发市场和社会活力。一是深化行政管理体制改革。以更大气魄简政放权，再取消、下放和调整一批行政审批事项，全面清理非行政许可，大幅减少前置审批。规范行政审批管理，完成政府部门权力清单、责任清单公布工作，推广网上并联审批等新模式，既要从越位点退出，也要把缺位点补上。对应该放给社会和企业的权力，要松开手、不截留，强化事中事后监管。二是深化投融资体制改革。落实投融资机制改革措施，推动非公资本参与国企项目建设，积极推行政府和社会资本合作融资模式。健全金融为实体经济服务的体制机制，加快发展服务小微企业的中小金融机构。三是深化财税体制改革。认真贯彻预算法，实行全面规范、公开透明的预算管理制度，凡使用政府资金的部门，除涉密信息外都要公开预决算。进一步拓展“营改增”实施范围。四是健全社会信用体系。着力推进政务诚信、商务诚信和社会诚信体系建设，褒扬诚信，惩戒失信，营造良好的信用环境。五是深化国资国企改革。完善国有资产管理体制，以管资本为主加强国有资产监管，改革国有资本授权经营体制，创新国有资本运营机制。推动国有企业完善现代企业制度，适应市场化、国际化新趋势，以规范经营决策、资产保值增值、公平参与竞争、提高企业效率、增强企业活力、承担社会责任为重点，进一步深化国有企业改革。六是大力发展混合所有制经济和非公经济。推动国有资本、非公有资本等交叉持股、融合发展。推进混合所有制企业实行管理人员、技术骨干和员工持股，形成资本所有者和劳动者利益共同体。废除各种不合理规定，消除隐性壁垒，为非公经济发展创造宽松环境，鼓励有条件的非公企业建立现代企业制度，引导非公资本控股混合所有制企业。七是扎实推进公务用车制度改革。

　　（二）加大开放力度，再创竞争优势。开放是大连的最大优势所在。抓住国家实施“一带一路”战略的有利机遇，增强对外开放的主动性、引领性，实现更高水平的对外开放。借鉴上海自贸区的经验，积极推动制度创新，通过放管结合的改革，提高贸易便利化水平，营造国际化、法治化营商环境。加大力度推进金普新区开发建设，按照完善规划、理顺体制、创新机制、强化功能的要求，先行先试，大胆实践，加快“七通一平”建设，加速产业集聚，有序推进新区的建设和发展，力争3年初见成果、5年大见成效。发挥大连、长兴岛和旅顺3个国家级经济技术开发区的功能作用，科学进行产业定位，加大招商引资力度，使之成为国际生产要素集聚配置的高地。提升保税区和保税港区的功能，大力发展保税业务，不失时机地做好自由贸易园区申办工作。加快花园口经济区的开发开放步伐。积极推进中日韩循环经济示范园区建设。强化高新区的对外技术合作优势，引进和培育一批创新能力强的高新技术企业。提高利用外资质量，既要以软硬环境优势吸引国内外要素在我市配置，又要以实体经济存量引入外来要素混合发展，跟踪项目线索，争取更多项目签约落地，全年外商投资注册资本要达到27亿美元以上。精心筹备2015大连夏季达沃斯年会，发挥对外开放平台作用，向世界展示大连繁荣发展的崭新面貌。

　　（三）实施创新驱动，加快转型升级。以更大的勇气和决心，坚定不移走科技引领、创新驱动之路。打破制约科技与经济融合的体制机制障碍，以企业为主体、市场为导向、产学研用相结合，构建布局合理、功能完善、独具特色的创新体系。创建国家自主创新示范区，实施自主创新普适性优惠政策。加大对公共科技活动的支持力度，搭建开发共享平台。鼓励企业技术创新，从基础工艺、零部件和材料研发入手，实现产业链和技术链深度融合。强化企业市场意识和创新意识，完善技术转移和金融支持体系，激发企业承接科技成果转化的内在需求。加速优势产业与移动互联的融合发展。大力培育创新主体和新兴业态，形成一批创新型龙头企业和创新型中小企业集群。推进人才政策创新，增强城市对人才的吸引力。继续实施海外高层次人才引进工程、领军人才培养工程和专业技术人才知识更新工程，为经济社会发展提供人才支撑。

　　（四）建设法治政府，推进依法治市。全面贯彻党的十八届四中全会精神，落实市委的决策部署，自觉在法治轨道上开展工作，加快建设职能科学、权责法定、执法严明、公开公正、廉洁高效、守法诚信的法治政府。一是依法履行政府职能。坚持法无授权不可为、法定职责必须为，建立行政机关敢于担当、敢于负责的激励评价机制。二是完善科学决策程序。把公众参与、专家论证、风险评估、合法性审查、集体讨论决定作为重大行政决策的法定程序，确保行政决策制度科学、程序正当、过程公开、责任明确。三是深化行政执法体制改革。科学划分市、区（市、县）政府的事权和职能，创新执法体制，完善执法程序，推进综合执法，严格执法责任，建立权责统一、权威高效的依法行政体制，按照减少层次、整合队伍、提高效率的原则，合理配置执法力量，实行执法人员持证上岗，落实资格管理制度，做到严格、规范、公正、文明执法。四是强化行政权力监督。加大行政监察和审计监督力度，全面推进政务公开，自觉接受人大及其常委会法律监督、工作监督和政协的民主监督，认真执行人大及其常委会决议决定，做好意见、建议和提案办理工作，广泛听取民主党派、工商联、无党派人士、各人民团体的意见，高度重视社会监督和新闻舆论监督，让权力在阳光下运行。

　　（五）转变工作作风，提高政府效能。新形势新任务对政府机关及其工作人员提出了更高的要求，我们要开阔视野、提升本领、崇尚实干、勇于创新，循规律、出对策、施实招。积极开展“工作创新年”活动，创新理念，创新思路，创新方法。以创新精神破解难题、推动发展。巩固党的群众路线教育实践活动和“四风”整治成果，进一步落实整改措施，强化服务意识，提高行政效能。深化政风行风建设，严肃查处影响制约科学发展和损害群众利益的违纪违法案件。严格控制“三公”经费开支，厉行节约，艰苦奋斗，树立清正廉洁的良好形象。坚决纠正不作为、乱作为，坚决惩处失职、渎职行为。强化工作落实责任制，凡是部署了的工作、明确了的任务，必须不折不扣落实到位。加强督促检查，定期通报工作任务落实情况，确保全年任务目标顺利完成。

　　各位代表，2015年改革发展任务异常艰巨而繁重，人民群众期盼着城市更美、生活更富、工作学习更舒心。我们要用恪尽职守诠释责任，用奋发有为回应期待，通过一个个工程的实施、一项项任务的完成、一件件实事的落实，赢得人民群众的支持和拥护，再创人民满意的新业绩！

　　各位代表，新的征程已经开启。让我们紧密团结在以习近平同志为核心的党中央周围，在市委的坚强领导下，振奋精神，坚定信心，真抓实干，砥砺奋进，共同创造大连更加美好的明天！
